# Childhood-Onset Systemic Lupus Erythematosus Presenting With Concomitant Gastrointestinal Manifestation and Antiphospholipid Syndrome

**DOI:** 10.7759/cureus.49205

**Published:** 2023-11-21

**Authors:** Nur Diyana Mohd Shukri, Wan Majdiah Wan Mohamad, Wan Suriana Wan Ab Rahman

**Affiliations:** 1 School of Medical Sciences, Universiti Sains Malaysia, Kubang Kerian, MYS; 2 School of Dental Sciences, Universiti Sains Malaysia, Kubang Kerian, MYS

**Keywords:** autoimmune disease, systemic lupus erythematosus, antiphospholipid syndrome, gastrointestinal manifestation, childhood-onset sle

## Abstract

Childhood-onset systemic lupus erythematosus is a rare disease that is more prevalent in Southeast Asian children than in Western children. It is characterised by a peripubertal onset and a female predominance that rises with age. Haematological, renal, and mucocutaneous are among the frequently involved organs upon diagnosis. Some of the typical symptoms include cutaneous vasculitis, malar rash, and fever. Patients frequently had proliferative class IV lupus nephritis, which increases disease activity and kidney damage. We reported a child presented with fever associated with multiple joint pain, skin rashes over the fingers of the right hand, and generalised abdominal pain.

## Introduction

Systemic lupus erythematosus (SLE) is a chronic systemic autoimmune disease with variable clinical symptoms and severity [1]. Childhood-onset systemic lupus erythematosus (cSLE) is a term used to refer to individuals who developed SLE before the age of 18 years old [2]. The median age of onset for cSLE is between 11 and 12 years with more severe disease at presentation, and it rarely affects children less than five years old [3].

Common presentations in children are fever, malar rash, and cutaneous vasculitis [1]. In Malaysia, a study by Lim et al. reported common presentations of Malaysian children with SLE are fever, vasculitis rash, and lethargy with predominant cutaneous lupus, leucopoenia, thrombocytopenia and significant renal, liver, and central nervous system involvement [4]. We report a case of childhood-onset SLE who presented with gastrointestinal manifestation and concomitant antiphospholipid syndrome, which has rarely been reported before. This case highlights the different and rare clinical presentations that can occur in paediatric SLE patients.

## Case presentation

This is a 13-year-old Chinese girl, with no underlying medical illness, presented with a preceding history of low-grade fever for one month associated with multiple joint pain involving small joints of the hands, shoulder, and knee joints. She also complained of multiple spots of skin rashes over the fingers of her right hand, generalised abdominal pain, loss of appetite, and loss of weight. She denied a history of significant hair loss, oral ulcer, photosensitivity, malar rash, nausea, or vomiting.

During admission, her vital signs were normal with good hydration status and afebrile. There were rashes over her right hand suggestive of vasculitis. Abdominal examination revealed soft and tender, with mild hepatomegaly. However, there were no oral ulcer, palatal ulcer, discoid rash, and splenomegaly. Examination of other systems was unremarkable.

Initial blood workup showed pancytopenia with elevated liver enzymes (Table 1). Renal function test results were normal. Urine examination showed the presence of haematuria. Autoimmune disease screening was positive for anti-nuclear antibodies (ANA), anti-double-stranded DNA (anti-dsDNA), and extractable nuclear autoantibodies (ENA). Complement C3 and C4 levels were also decreased. Increased levels of anticardiolipin antibodies and beta2-glycoprotein-1 antibodies suggested the presence of underlying antiphospholipid syndrome (APS) in SLE. A renal biopsy was done, and the result revealed interstitial nephritis with diffuse lupus nephritis class IV. Ultrasound abdomen revealed bilateral parenchymal renal changes. There was no liver pathology identified despite the presence of hepatomegaly. The Systemic Lupus Erythematosus Disease Activity Index 2000 (SLEDAI-2K) score demonstrated high disease activity status signifying a more severe disease.

**Table 1:** Relevant investigation results of the patient ANA: anti-nuclear antibodies, anti-dsDNA: anti-double-stranded DNA, ENA: extractable nuclear autoantibodies, anti-SmD1: anti-smith antibodies directed to D1 protein, anti-Ku: anti-Ku antibodies, anti-U1-snRNP: anti-U1-small nuclear ribonucleoprotein, anti-PO/RPP: anti-ribosomal P proteins (RPP) designated PO (38 kd), anti-SS-A/R0 60 kD: anti-Sjogren's syndrome type A targeting the Ro 60 kD protein, SS-B/La: Sjogren's syndrome type B, GPL: IgG phospholipid units, MPL: IgM phospholipid units, ESR: erythrocyte sedimentation rate, AST: aspartate aminotransferase, ALP: alkaline phosphatase, ALT : alanine transaminase, WBC: white blood cell, Hb: haemoglobin, PT: prothrombin time, APTT: activated partial thromboplastin time, INR: international normalised ratio, RBC: red blood cell, Retic: reticulocyte.

Investigation		Result	Reference
Immunological			
ANA		Positive 1:320 Homogenous pattern	-
Anti-dsDNA		>400 IU/ml	
ENA		Positive anti-SmD1, anti-histone, anti-nucleosome, anti-Ku, anti-U1-snRNP, anti-PO/RPP, Anti-SS-A/R0 60 kD, anti-SS-B/La	
Complement C3		0.27	0.66-1.3 g/L
Complement C4		0.03	0.2-0.6 g/L
Beta2 glycoprotein I	IgG	236.82	>18 U/ml
	IgM	57.73	>18 U/ml
Anti-cardiolipin antibodies (ACA)	IgG	39.31	<10 GPL
	IgM	14.07	<7 MPL
Infective			
ESR		122 mm/H	-
C-reactive protein		Negative	-
Biochemical			
Liver function test	Albumin	26	38-44 g/L
	AST	423	5-34 U/L
	ALP	200	<406 U/L
	ALT	191	13-45 U/L
Renal function test	Urea	8.2	1.7-8.3 mmol/L
	Sodium	138	135-145 mmol/L
	Potassium	4.5	3.5-5.0 mmol/L
	Creatinine	93	70-130 µmol/L
Haematological			
Full blood count	WBC	4.69	3.4-10.1 x 10^9^/L
	Hb	5.9	11.6-15.1 g/dl
	Platelet	98	158-410 x 10^9^/L
	Lymphocyte numbers	0.88	x 10^9^/L
	Retic	4.11%	0.48%-1.48%
Coagulation test	PT	13.4	12.61-15.72 sec
	APTT	84.8	30.0-45.8 sec
	INR	1.03	0.86-1.14
Full blood picture		Severe anaemia with RBC features suggestive of haemolysis and thrombocytopaenia	
Imaging			
Ultrasound abdomen		Bilateral renal parenchymal changes. Bilateral pleural effusion, left more than right	

She was diagnosed to have childhood-onset SLE with lupus nephritis and was given prednisolone and hydroxychloroquine for two months. Repeated blood tests showed normalised liver enzymes might suggest elevated liver enzymes most probably due to the intake of medications. Her condition improved well after treatment with oral prednisolone and hydroxychloroquine.

## Discussion

SLE is a chronic, systemic autoimmune disease involving multiple organ systems which commonly involves renal, haematological, cutaneous, musculoskeletal, and neurological systems [5]. SLE patients usually presented with a wide range of clinical manifestations. Gastrointestinal manifestations in childhood with SLE are considered atypical and reported in 20% of the cases. The common presenting symptoms include abdominal pain, nausea, vomiting, diarrhoea, and gastrointestinal bleeding [3].

Abdominal pain in SLE may be due to the lupus involvement itself [6]. However, other medical conditions like viral or bacterial infections, side effects of steroid medications, or medical emergency such as intestinal perforation need to be excluded first. Acute abdomen should be treated promptly as it can be life-threatening in case of acute pancreatitis, perforated viscous, or bowel ischemia. Less common gastrointestinal manifestations of childhood SLE include hepatitis, lupus peritonitis, and lupus acalculous cholecystitis [6].

Gastrointestinal manifestations in children can vary from the most common presentation of lupus mesenteric vasculitis to the rare one such as intestinal pseudo-obstruction with a prevalence rate of 1.3% [7]. Previous studies had reported gastrointestinal manifestation of cSLE presenting with SLE-related pancreatitis and lupus enteritis [8,9].

Blood investigations revealed she had pancytopenia. These findings are in accordance with a retrospective multi-centre study by Akca et al. [10], who reported that anaemia (n=88, 74.5%) was the most prevalent hematologic abnormality, with autoimmune haemolytic anaemia accounting for the majority of cases (n=40). Additionally, a study by Tan et al. found that the majority of patients (81.3%) had lymphopenia at presentation indicating the usefulness of this finding in considering the differential diagnosis of SLE in children [11].

Positive ANA, anti-dsDNA, and ENA support the diagnosis of SLE. Her ENA was positive for most of the autoantibodies including anti-smith antibodies directed to D1 protein (anti-SmD1), anti-histone antibodies (anti-histone), anti-nucleosome, anti-Ku antibodies (anti-Ku), anti-U1-small nuclear ribonucleoprotein (anti-U1-snRNP), anti-ribosomal P proteins (RPP) designated PO (38 kd) (anti-PO/RPP), anti-Sjogren's syndrome type A targeting the Ro 60 kD protein (anti-SS-A/R0 60 kD), and anti-Sjogren's syndrome type B (anti-SS-B/La). The decrease in C3 and C4 levels indicates the presence of complement consumption. The deficiencies of early components of the classic complement pathway contribute to the onset of SLE at a young age [12]. Low C3 level was significantly found more frequent in patients with haematological involvement (p=0.008) [10]. Increased levels of anti-cardiolipin antibodies and Beta2-glycoprotein-1 antibodies suggest the presence of underlying APS in SLE. Similar findings had been reported by Akca et al. with the presence of anti-cardiolipin antibodies (p=0.001), beta2-glycoprotein-1 antibodies (p≤0.001), and anti-Smith antibody positivity in their study [10].

She was diagnosed as active SLE as she fulfilled the Systemic Lupus International Collaborating Clinics (SLICC) criteria [13]. According to these criteria, the patient should fulfil at least four criteria, including at least one each from the clinical and laboratory levels, or biopsy-proven lupus nephritis with a positive ANA or anti-DNA test. This patient fulfilled nine out of 17 criteria (arthritis, renal involvement, lymphopenia, thrombocytopenia, positive ANA, ds-DNA, anti-Smith antibody, anti-phospholipid antibody, and low C3 and C4 levels).

Antiphospholipid syndrome (APS) is an acquired systemic autoimmune disease characterized by arterial or venous thrombosis, pregnancy-related morbidity, and persistent anticardiolipin antibodies. It can occur as primary APS or secondary to autoimmune diseases such as SLE. In this case, the patient is having concomitant SLE with APS which might impose a great consequence to the disease progression and multiple organ damage. Unlike APS in adults, APS in children is rare and not completely understood [14]. The most possible cause of abdominal pain in this case might be due to the presence of mesenteric vein thrombosis. In accordance with Madison et al., the presence of anticardiolipin antibodies increases the risk for arterial and venous thrombosis in children [14].

In childhood-onset systemic lupus erythematosus, renal involvement is more common in children than in adults and is associated with considerable morbidity and mortality. Class IV lupus nephritis is the predominant histopathology of lupus nephritis in Asian children (39.4% to 54.0%) [1]. Studies showed that children usually presented with a more severe renal manifestation at diagnosis. Renal involvements can be silent and range from mild nephritis to overt renal failure. However, some patients were asymptomatic. In this case, a renal biopsy was performed and confirmed the diagnosis of lupus nephritis.

## Conclusions

The presenting features in cSLE may be diverse. Atypical presentation may be associated with comorbidities and cause a delay in diagnosis. Autoantibody profiles may help in predicting the clinical course of the disease and identify patients at risk of developing major organ involvement. However, since this is a single case report, larger studies with more sample sizes are required to obtain a stronger justification. Nevertheless, increasing awareness towards the disease and proper assessment are vital to achieve an accurate diagnosis and prevent further complications of the disease.
